# Commentary: Undifferentiated pleomorphic sarcoma of the adrenal gland: a case report and literature review

**DOI:** 10.3389/fonc.2026.1685761

**Published:** 2026-02-05

**Authors:** Tomonori Kawasaki, Takuya Watanabe, Masataka Hirasaki, Satoshi Kanno, Ryuzo Kawamura, Arisa Kokubo, Shinji Furuya, Masanori Wako, Jiro Ichikawa

**Affiliations:** 1 Department of Pathology, Saitama Medical University International Medical Center, Hidaka, Japan; 2 Department of Orthopaedic Oncology, Saitama Medical University International Medical Center, Hidaka, Japan; 3 Department of Clinical Cancer Genomics, Saitama Medical University International Medical Center, Hidaka, Japan; 4 Department of Chemistry, Saitama University, Sakura, Japan; 5 First Department of Surgery, Interdisciplinary Graduate School of Medicine, University of Yamanashi, Chuo, Japan; 6 Department of Orthopaedic Surgery, Interdisciplinary Graduate School of Medicine, University of Yamanashi, Chuo, Japan

**Keywords:** dedifferentiated liposarcoma, exclusion diagnosis, histopathology, leiomyosarcoma, rhabdomyosarcoma, undifferentiated pleomorphic sarcoma

## 1 Introduction

Undifferentiated pleomorphic sarcoma (UPS), previously known as a malignant fibrous histiocytoma, is a type of pleomorphic sarcoma, including dedifferentiated liposarcoma and pleomorphic leiomyosarcoma. Because sarcomas originating in the adrenal gland are extremely rare and this case is exceptionally intriguing, we were interested in the recent publication by Xiaochuan et al. entitled “Undifferentiated pleomorphic sarcoma of the adrenal gland: a case report and literature review” (1), and express our gratitude to the authors for reporting this invaluable case. However, UPS excluded differential diagnoses (2). Here, we discuss the key differential diagnoses in this case and provide recent insights into UPS.

Subsections relevant for the subject.

First, regarding imaging findings, this study presents only plain computed tomography images, making it impossible to strictly evaluate the internal characteristics of the tumor. Therefore, we discuss the differential diagnoses that should be excluded in diagnosing UPS.

## 2 Commentary and discussion

### 2.1 UPS

Pathological findings are crucial for the diagnosis of UPS. Key points include the lack of specific markers detectable by immunohistochemistry (IHC) or Fluorescence *in situ* hybridization (FISH) in UPS and necessity of excluding other spindle cells and pleomorphic sarcomas (2). Specific diagnostic considerations include 1) the possibility of pleomorphic sarcomas with differentiation along specific lines, 2) presence of dedifferentiated sarcoma components, and 3) potential for sarcomatoid features in the carcinoma (3). Specifically for (1), the absence of histological or IHC findings typically observed in other pleomorphic sarcomas must be demonstrated. Therefore, we aimed to introduce the clinical features of UPS (3). Surgical resection is the primary treatment for this condition. However, opinions on the effect of surgical margins on local recurrence and overall survival (OS) (4). In Xiaochuan, a margin of approximately 5 cm is considered safe (1). Nevertheless, UPS, as well as myxofibrosarcoma (MFS), are recognized as an infiltrative sarcoma that requires pathological evaluation of surgical margins because the recurrence rate in cases of infiltrative UPS and MFS increases as the distance of the tumor margin becomes shorter (4). Chemotherapy and radiotherapy are often combined with surgery. Neoadjuvant chemotherapy for UPS improved raplase-free survival (RFS) and OS. Adjuvant chemotherapy significantly extended OS in patients with localized UPS and was identified as an independent prognostic factor. The current regimen includes single or combination therapies with ifosfamide and anthracyclines, whereas combinations, such as gemcitabine and docetaxel, molecular targeted therapies, and immunotherapies, are under investigation with expected results (5). Radiotherapy is frequently used, with postoperative radiotherapy reported to significantly improve local RFS and OS (6).

### 2.2 Important differential diagnoses for UPS

#### 2.1.1 Dedifferentiated liposarcoma

The histological features of dedifferentiated liposarcoma (DDLPS) involve progression from atypical lipomatous tumor/well-differentiated liposarcoma to high-grade nonlipogenic sarcomas. Dedifferentiated components exhibit a wide morphological spectrum and often resemble UPS or MFS in histological patterns (7). Morphologically, they show moderate or high cellularity, moderate to marked pleomorphism, and the cells are arranged in patternless distributions, loose fascicles, or storiform “malignant fibrous histiocytoma-like” architecture (8). Moreover, approximately 5–10% display heterologous differentiation, commonly involving muscle, bone, or cartilage differentiation, with particular attention required for differentiation from leiomyosarcoma or rhabdomyosarcoma if muscle differentiation is present (9). IHC and FISH findings are crucial for overcoming challenges in histological diagnosis. In DDLPS, markers, such as CD34, α-SMA, and desmin, may be positive, with the expression of MDM2 and CDK4 being significant. The combination of MDM2, CDK4, and p16 is frequently used (8). Amplification of MDM2 via FISH is commonly confirmed with a positivity rate > 90% (10). However, in UPS cases involving the retroperitoneum and thigh, IHC showed positivity for MDM2 in 22%, CDK4 in 29%, and both in 18% of the cases. Additionally, amplification of MDM2 and/or CDK4 via FISH has been observed in 13% of UPS cases (11). Therefore, differentiating DDLPS from UPS requires a comprehensive diagnosis that combines morphological observations with IHC and FISH analyses.

#### 2.1.2 Leiomyosarcoma

Leiomyosarcoma (LMS) is a malignant neoplasm comprising cells with smooth muscle differentiation, and many of these tumors are highly malignant (12). Among these, pleomorphic LMS (PLMS) is particularly difficult to differentiate from UPS. PLMS is characterized by older age at presentation, deep limb or retroperitoneal origins, and frequent metastases (13). As for IHC markers, LMS overall can be diagnosed using α-SMA, desmin, and h-caldesmon, with at least one of these markers being positive in 100% of cases and > 70% of cases showing positivity for ≥ 2 markers. However, no specific markers have been identified. Cytokeratin, EMA, and CD34 may be positive, and are often focal. These expressions generally hold true for PLMS, although in 50–70% of cases, at least one myogenic marker is positive; however, compared to typical LMS, these markers are often weakly stained or focal in PLMS (12). LMS has been reported to metastasize at 29–44%, with a 10-year survival rate of only 49–60% (14). PLMS demonstrates a high proliferative capacity and aggressive biological behavior, making careful postoperative follow-up essential (15).

#### 2.1.3 Rhabdomyosarcoma

Among its subtypes, pleomorphic rhabdomyosarcoma (PRMS) is particularly challenging to differentiate from the UPS. Although RMS predominantly affects children, PRMS is more commonly found in the elderly and often arises in the deep soft tissues of the extremities (13). PRMSs comprise sheets of atypical, large, and frequently multinucleated polygonal, spindle-shaped, or rhabdoid cells (16). In IHC, desmin is typically positive, whereas the expression of MyoD1 and myogenin is often limited; however, these findings are essential for distinguishing PRMS from UPS (17). PRMS is a highly aggressive sarcoma with a mean survival of 7.3 months, and 80% of patients reportedly succumb to this disease (16).

## 3 Conclusion

Here, we examined the differential diagnoses that should be considered in the diagnosis of UPS and an overview of UPS itself. In diagnosing UPS, morphological features and IHC marker expressions should be presented while emphasizing the concept that “exclusion diagnosis is principle.
